# Re-emerging Tularemia in Some Middle East Countries: What Are the Reasons?

**Published:** 2018-02

**Authors:** Shirin SHAHSAVARI, Hossein BANNAZADEH BAGHI, Hossein SAMADI KAFIL, Hamed EBRAHIMZADEH LEYLABADLO

**Affiliations:** 1. Division of Biotechnology, Dept. of Pathobiology, Faculty of Veterinary Medicine, Ferdowsi University, Mashhad, Iran; 2. Infectious and Tropical Diseases Research Center, Tabriz University of Medical Sciences, Tabriz, Iran; 3. Dept. of Microbiology, School of Medicine, Tehran University of Medical Sciences, Tehran, Iran

## Dear Editor-in-Chief

In recent years, the re-emergence of tularemia, an acute zoonotic disease, has been reported in many Middle Eastern countries such as Turkey and Iran (1). Human travel, animal or birth migration, warfare, climate variations and natural calamities are several factors that can have an effect on the relationship between human, parasites, and vectors or rodents and play a key role in the circulation of infections. Natural disasters, such as earthquake, are one of the main examples in the spread of infectious diseases, because it endangers public health, changes ecosystems, decreases access to clean water and safe food. However, human and infected animals or vector contingencies are increasing (2, 3).

Recently, several tularemia re-emergence cases have been reported in Iran. After an earthquake, in the Sistan and Baluchestan Province, serum agglutination test in rodents showed signs of tularemia and tularemia vectors were found on these rodents. This is believed to have happened because after disasters happen rodents have to leave their safe shelters and pose as high-risk infection sources for humans (4). Another survey, on serological studies among butchers and slaughterhouses in this area, eventuated that tularemia is endemic in the Sistan and Baluchestan Province in southeastern Iran, but no evidence for the increase in seroprevalence of infection in aged patients was found that might suggest that the infection is re-emerged. The reason for this outbreak was that butchers and slaughterhouse are in the high-risk group for tularemia infections exposed to *Francisella tularensis* because of their direct contact with infected meat or animals during their work (5). Afghanistan, one of the neighboring countries in this area, also deals with unsafe health conditions and potential resource of tularemia vectors. Due to uncontrolled animal and human traffic between the borders of these areas, the Sistan-Baluchestan Province isolated in a very high-risk environment (4). Investigations into tularemia in the western Iranian province of Kurdistan showed the highest amounts of seroprevalence in hunters with direct contact with foxes. The seroprevalence of infection among individuals are living in this area hence is quite high, because the consumption of hunted meat in this area is very common compared with other regions of Iran (6).

Oropharyngeal tularemiais the most common form of tularemia infections in Turkey and epidemiological studies emphasize the seasonal manner since it mainly spreads during the rainy season in winter and in endemic regions where aquatic cycle is common. After rainfall, the infections usually circulate by infected aquatic animal urine or feces and pollutes the surface of waters and water sources (2, 7). Outbreaks are prevalent in areas consuming contaminated drinking tap water. In these regions, dysfunction of chlorination devices for water reservoir tanks, no piped reservoirs inlet supplying and the uncovered waterways, that exposure water to infections and also the rodents activity(that are effective in tularemia cycle) near the surface of water storage tankare the most important reasons (8, 9).

Many strategies such as monitoring the health of travelers or animals on borders with countries that are reservoirs of infectious diseases and prohibiting the use of meat with questionable health status can prevent the transmission of tularemia greatly. Since the Oropharyngeal tularemia is a water-borne disease, in endemic areas, use of chlorinated water must be considered.
